# Autoclave mediated one-pot-one-minute synthesis of AgNPs and Au–Ag nanocomposite from *Melia azedarach* bark extract with antimicrobial activity against food pathogens

**DOI:** 10.1186/s13065-016-0157-0

**Published:** 2016-03-31

**Authors:** Alok Pani, Joong Hee Lee, Soon-II Yun

**Affiliations:** Department of Food Science and Technology, College of Agriculture and Life Sciences, Chonbuk National University, Jeonju, 561-756 Republic of Korea; Department of BIN Convergence Technology, Chonbuk National University, Jeonju, 561-756 Republic of Korea

**Keywords:** One-pot-one-minute, AgNPs, Au–Ag nanocomposite, Autoclave, Green synthesis, Galvanic replacement

## Abstract

**Background:**

The increasing use of nanoparticles and nanocomposite in pharmaceutical and processed food industry have increased the demand for nontoxic and inert metallic nanostructures. Chemical and physical method of synthesis of nanostructures is most popular in industrial production, despite the fact that these methods are labor intensive and/or generate toxic effluents. There has been an increasing demand for rapid, ecofriendly and relatively cheaper synthesis of nanostructures.

**Methods:**

Here, we propose a strategy, for one-minute green synthesis of AgNPs and a one-pot one-minute green synthesis of Au-Ag nanocomposite, using *Melia azedarach* bark aqueous extract as reducing agent. The hydrothermal mechanism of the autoclave technology has been successfully used in this study to accelerate the nucleation and growth of nano-crystals.

**Results:**

The study also presents high antimicrobial potential of the synthesized nano solutions against common food and water born pathogens. The multistep characterization and analysis of the synthesized nanomaterial samples, using UV-visible spectroscopy, ICP-MS, FT-IR, EDX, XRD, HR-TEM and FE-SEM, also reveal the reaction dynamics of AgNO3, AuCl3 and plant extract in synthesis of the nanoparticles and nanocomposite.

**Conclusions:**

The antimicrobial effectiveness of the synthesized Au-Ag nanocomposite, with high gold to silver ratio, reduces the dependency on the AgNPs, which is considered to be environmentally more toxic than the gold counterpart. We hope that this new strategy will change the present course of green synthesis. The rapidity of synthesis will also help in industrial scale green production of nanostructures using *Melia azedarach*.

## Background

Colloids and interface have been the cause of many natural phenomena since time immemorial. The dynamics of colloids was first described by Albert Einstein, in his dissertation, by the term Brownian motion [1]. Nano-scale metal, which also exhibits the colloidal properties, was first described scientifically by Michel Faraday in optical terms [2]. Since then many people in the scientific community have tried-and-succeeded in synthesizing metal nanostructures by seeded and non-seeded attempt [3–5]. The metal nanostructures reported till today have been synthesized physically, chemically or biologically [6–11]. The most popular are the synthetic nano products which are synthesized for specific usage in optical, electrical and mechanical fields [12–16].

Until recently, the mainstream nanostructure production has been dominated by chemicals, for faster and uniform synthesis, and/or is very labor intensive [17]. Due to the chemical genesis of nanostructures, the residual chemical components within the nanostructures pose a major toxicity risk at various concentrations in the environment and during bio-applications [18, 19]. These setbacks groomed the scientific minds around the world to use the biological systems and bio-products as a preferred and effective substitute for the clean and green synthesis of biocompatible nanostructures [10].

Most of the metal nanostructure research has been concentrated on the synthesis of noble metal nanoparticles such as gold and silver [10, 20]. The plant extract based synthesis of gold and silver nanoparticles with antimicrobial and biocompatibility properties has been a success [21, 22]. Although plant extracts based methods have shown success in faster synthesis of gold and silver nanostructures but it is not fast enough to compete with the chemical methods [23, 24].

In this article, we introduce a strategy, for one-minute green synthesis of AgNPs and a one-pot one-minute green synthesis of Au–Ag nanocomposite, using *Melia azedarach* bark aqueous extract as reducing agent. The bark extract is known to contain phytochemicals such as triterpenoids, flavonoids, glycosides steroids and carbohydrates [25]. It is also known to contain polyphenolic compounds, resulting in high antioxidant activity [26]. The synthesis of silver nanoparticles is based on the concept of thermal decomposition of silver nitrate, in the presence of a reducing agent. Whereas, the integrated strategy behind the one-pot one-minute synthesis of Au–Ag nanocomposite comprises (1) The bio-thermal reduction of silver nitrate to silver nanoparticles, and (2) the galvanic displacement reaction of auric chloride with the silver nanoparticles. Here we have used the autoclave technology, invented by Charles Chamberland in 1879, to generate the required amount of heat and pressure. The autoclave technology has been used to grow synthetic quartz crystals and to cure composites [27, 28]. The controlled environment provided by the autoclave ensures that the best possible physical properties are reputably attainable and repeatable. So, the hypothesis is that the hydrothermal energy (121°C, 15 psi) generated by an autoclave, for 1 min, is enough to accelerate the metal reduction capacity of the plant extract. There have been some studies, in the recent past, in favor of this hypothesis, but the synthesis time has been restricted to 5 min. [29, 30].

Here we also present a comparative analysis of antimicrobial potential of the synthesized AgNPs and Au–Ag nanocomposite on six diverse food born pathogens. The synthesized nanoparticles and nanocomposite were passed through a multi-technique characterization to prove the authenticity of their quality and quantity.

## Results and discussion

The primary focus of this study is to prove the successful working of the proposed ecofriendly strategy for 1 min green synthesis of the nanostructures and to explain the reaction dynamics of silver nitrate, auric chloride and plant extract, during rapid synthesis of AgNPs and Au–Ag nanocomposite, using autoclave technology. The secondary focus was to analyze the antimicrobial activity of the synthesized nano solutions.

### Synthesis mechanism

To assess how conditions like high pressure and temperature affects the rate of synthesis of nanoparticles, samples were prepared by mixing metal salts to plant extract to make concentrations of 1, 5, 10 and 15 mM. The mixtures were then autoclaved for 1 min in a pre-heated (~110 °C) autoclave. After autoclaving the mixture containing silver salts showed different shades of brown, for different concentrations, which is a classic color of silver nanoparticles. During autoclaving silver nitrate undergoes thermal decomposition to give elemental silver [31]. The reaction dynamics of silver nitrate with plant extract can be represented by the following equation:

$${\text{2AgNO}}_{{\text{3}}} \left( {\text{s}} \right)\,+\, {\text{ Plant Extract }}\left( {{\text{aq}}} \right)
\mathop {\longrightarrow}\limits^{{~121^{\circ}{\text{C, 15 psi}}}}~{\text{2Ag }}\left( {\text{s}} \right) \,+\, {\text{ O}}_{{\text{2}}} \left( {\text{g}} \right) \,+\, {\text{ 2NO}}_{{\text{2}}} \left( {\text{g}} \right) \,+\, {\text{ Plant Extract }}\left( {{\text{aq}}} \right) $$

Au–Ag nanocomposite formed instantly when auric chloride was added to the freshly prepared silver nanoparticles. After autoclaving the temperature was immediately brought down to ~100 °C by releasing the pressure in the autoclave and auric chloride was mixed into the silver nano solution and was cooled at room temperature. These elemental silver particles thus generated get oxidized in the presence of oxygen and water [32]. As a result of oxidation the surface of the silver particles generates silver ions which dissolve in the water. The mixing of auric chloride to the brown color silver nano solution turned it into a brownish-violet solution in an instant. The mixing of auric chloride to the silver nano solution, at ~100 °C, initiates a galvanic replacement reaction [31].

$$ {\text{AuCl}}_{{4^{ - } }} \left( {\text{aq}} \right){ \,+\,}{\text{Ag}}\left( {\text{s}} \right) \to {\text{Au}}\left( {\text{s}} \right){ \,+ \, }{\text{Ag}}^+ \left( {\text{aq}} \right){ \,+\, }{\text{Cl}}^{-} \left( {\text{aq}} \right) $$

$$ 2 {\text{Ag }}\left( {\text{s}} \right)\text{ \,+\, } 2 {\text{H}}^{{ + }} \left( {\text{aq}} \right)\text{ \,+\, }{1 \mathord{\left/ {\vphantom {1 2}} \right. \kern-0pt} 2}{\text{O}}_{ 2} \left( {\text{aq}} \right) \to 2 {\text{Ag}}^{{ + }} \left( {\text{aq}} \right)\text{ \,+\, }{\text{H}}_{ 2} {\text{O}}\left( {\text{l}} \right) $$

As we should know that all material surfaces have some electrons from the environment but due to larger mass, the small amount of electrons on the surface become insignificant (e/m). Unlike most of the materials, the electrons gathered on the surface of nanoparticles becomes significant because of the low mass. Thus, in the present case scenario the galvanic displacement reaction cause the formation of Au particles bearing negative charge on the surface and the positively charged Ag ions. This results in formation of Au–Ag nanocomposite due to bonding of the Au nanoparticles and Ag ions.

The AgNp and Au–Ag nanocomposite solutions were kept at room temperature for further analysis and usage.

### Spectroscopic analysis

**Fig. 1:**
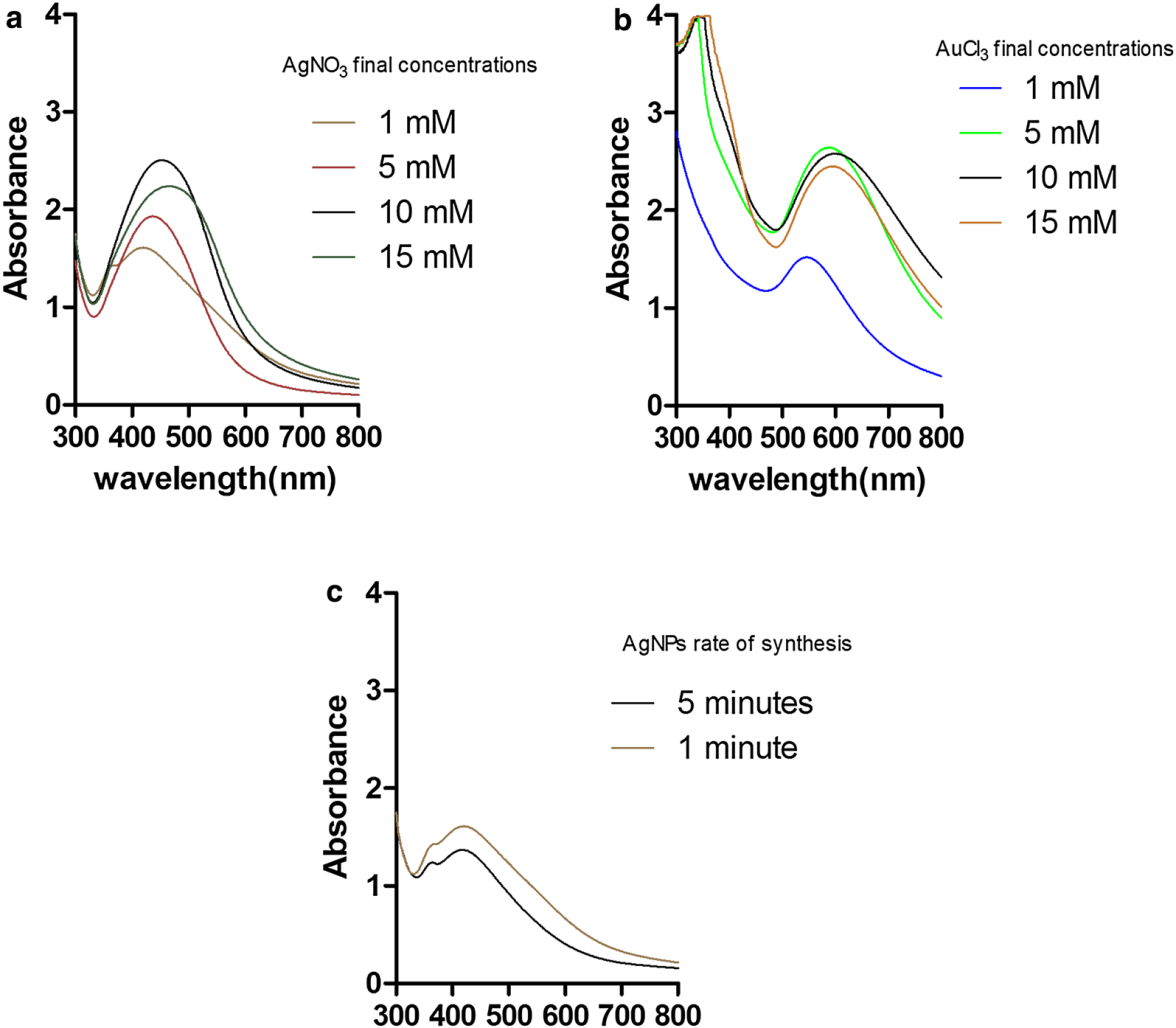
UV-visible spectra of **a** AgNPs synthesized by increasing concentration of AgNO_3_ in 10 ml plant extract, **b** Au–Ag nanocomposite synthesized by increasing concentration of AuCl_3_ in 10 ml 1 mM AgNPs (At ~ 100 °C), **c** AgNPs synthesized for different time period

After synthesis and cooling the AgNp and Au–Ag nanocomposite solutions to room temperature, 100 µl specimen of each solution was taken in a 96-well plate for UV-visible spectroscopy. The UV-visible spectra of AgNPs and Au–Ag nanocomposite has been shown in Fig. 1. The AgNPs spectral peak, of 10 mM AgNO_3_ concentration, exhibited highest absorbance at 440 nm (Fig. 1a). This gave the preliminary conformation of successful synthesis of AgNPs using the new method and the synthesis dynamics behind it. The Au–Ag nanocomposite spectral peak, of 5 mM AuCl_3_ concentration, on the other hand exhibited highest absorbance at 575 nm (Fig. 1b). In Fig. 1b, the peaks visible between 300 and 400 nm represent the very small silver nanoparticles formed after galvanic replacement and oxidation. The nanocomposite spectral peaks shows synthesis of AuNPs, which is a result of galvanic replacement reaction caused by the AgNPs present in the solution. The galvanic replacement reaction followed by oxidation of the silver nanoparticles with simultaneous interaction and encapsulation with the plant biomaterial, leads to the formation of a Au–Ag nanocomposite. Figure 1c shows that 1 min is just enough for complete synthesis.

Inductive coupled plasma mass spectroscopy (ICP-MS) was carried out to know the estimate concentration of Ag and Au particles in the synthesized solution. The concentration of Ag in 1 and 10 mM solution was 80 and 968 mg/l, respectively. The concentration of Au and Ag in Au–Ag nanocomposite solution (Containing 5 mM AuCl_3_ and 1 mM AgNO_3_) was 773 and 40 mg/l, respectively.

**Fig. 2:**
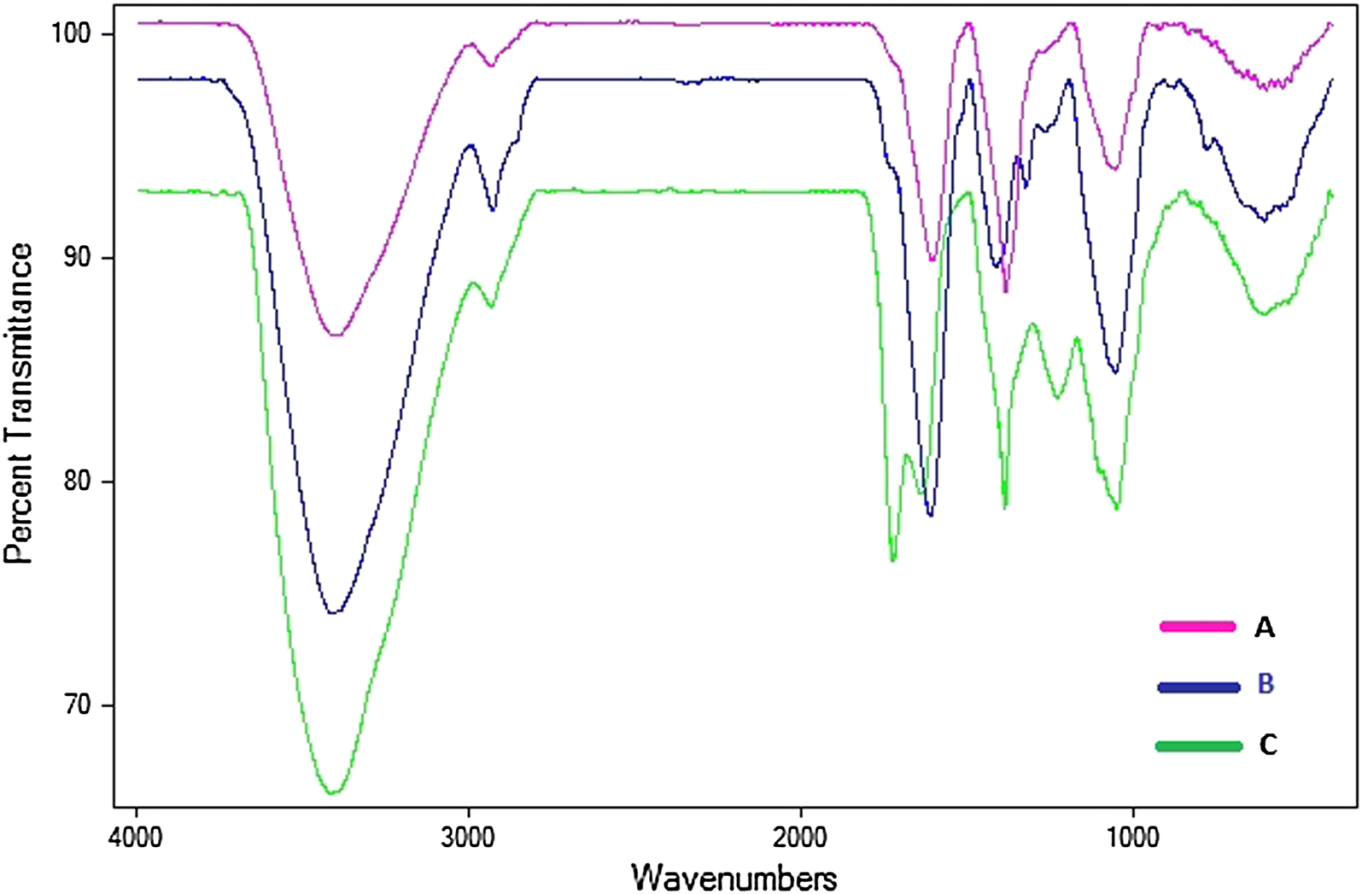
FTIR spectra showing the comparative vibrations and stretching of peaks of possible biomolecules present in the dried *A* AgNPs sample, *B* Plant extract sample and *C* Au–Ag nanoparticles sample

FTIR spectroscopy is generally used in green synthesis field to identify the possible biochemicals responsible for the synthesis and stabilization of the metal nano-structures. As the vibrational spectrum of a molecule is a unique physical property of the molecule, so the infrared spectrogram can be used as fingerprints of samples. The comparative view of the spectrograms of lyophilized bark extract, AgNPs and Au–Ag nanocomposite, shows a similarity in the absorption band pattern, which, confirms that the synthesis was from the bark extract and not only a physical process (Fig. 2).

If we compare the spectrograms of the bark extract, AgNPs and Au–Ag nanocomposite we can identify six major peaks showing vibrations and shift in wavenumbers (Fig. 2). The bark extract sample showed peaks at 3411 cm^−1^ (Hydroxy group, H-bonded OH stretch), 2927 cm^−1^ (Methylene C–H asymetric stretch), 1610 cm^−1^ (Conjugated ketone), 1412 cm^−1^ (Vinyl C–H in-plane bend), 1321 cm^−1^ (Carboxylate group) and 1053 cm^−1^ (cyclohexane ring vibrations) (Fig. 2b). Compared to the bark extract sample peaks, the AgNPs formed by reduction of Ag^+^ ions using the bark extract showed peaks at 3406 cm^−1^ (Hydroxy group, H-bonded OH stretch), 2930 cm^−1^(Methylene C–H asymetric stretch), 1603 cm^−1^ (Conjugated ketone), 1383 cm^−1^ (*gem*-Dimethyl stretch) and 1051 cm^−1^ (Cyclohexane ring vibration) (Fig. 2a). The Au–Ag nanocomposite mostly formed due to galvanic replacement showed peaks at 3415 cm^−1^ (Hydroxy group, H-bonded OH stretch), 2933 cm^−1^ (Methylene C–H asymetric stretching), 1721 cm^−1^ (ketone stretch), 1637 cm^−1^ (Conjugated ketone stretch), 1385 cm^−1^ (*gem*-Dimethyl/trimethyl stretch), 1230 cm^−1^ (Aromatic ethers, aryl-O stretch) and 1048 cm^−1^ (Cyclohexane ring vibration) (Fig. 2c). The absorption bands representing the hydroxy group, H-bonded OH stretch and the methylene C–H asymetric stretch are conjoined in all the three spectrograms, which suggest towards the presence of hydroxy methyle(CH_2_OH) group in the biomaterial. The gradual decrease in the intensity of the methylene band (B > A > C in Fig. 2), could be due to the loss of number of C–H bonds. The conjugated ketone band in the plant extract spectrogram remains unchanged in the AgNPs spectrogram but the Au–Ag nanocomposite spectrogram shows a split in the band, showing two peaks representing ketone and conjugated ketone. The main absorption band, of conjugated ketone, showing splitting of the absorption with change in relative band intensities could be due to the possible spatial/mechanical interaction of the adjacent carbonyl group with the addition of aqueous auric chloride to the AgNPs. The vinyl band is seen to be conjoined with a low intensity carboxylate band in the plant extract spectrogram. This suggest a possibility of vinyl carboxylate in the plant extract. Whereas, the AgNPs spectrogram shows an intense, gem-Dimethyl, band. Although the reaction is not very clear but this suggest that the interaction of silver nitrate with plant extract containing vinyl carboxylate gives gem-dimethyl. The band representing cyclohexane ring vibration remains unchanged during the AgNPs synthesis. On the other hand, the Au–Ag nanocomposite spectrogram clearly shows that the bands representing gem-dimethyl/trimethyl stretch, aryl-O stretch and cyclohexane ring vibration are joined together. This suggest the formation of aromatic ether compound with one aryl and one alkyl group. The aforementioned observation and analysis of the major absorption bands of the three spectrograms suggest an overall change of unsaturated compounds to saturated compounds in the biomaterial. This conversion of unsaturated to saturated, could be one of the reason for encaptulation of the nanomaterials.

It can be assuring from the spectrograms that there was no peak in the amide I and II regions, suggesting no microbial or fungal contamination in the sample. The peaks represent the functional groups present in the biomaterial, thus the steepness represents the difference in quantity [34]. A close comparision of the spectrogram shows that the peaks representing the functional groups were more sharper in the Au–Ag nanocomposite than the AgNPs, which suggest a better capping or adsorption of the biomaterial onto the surface of the nanocomposite.

**Fig. 3:**
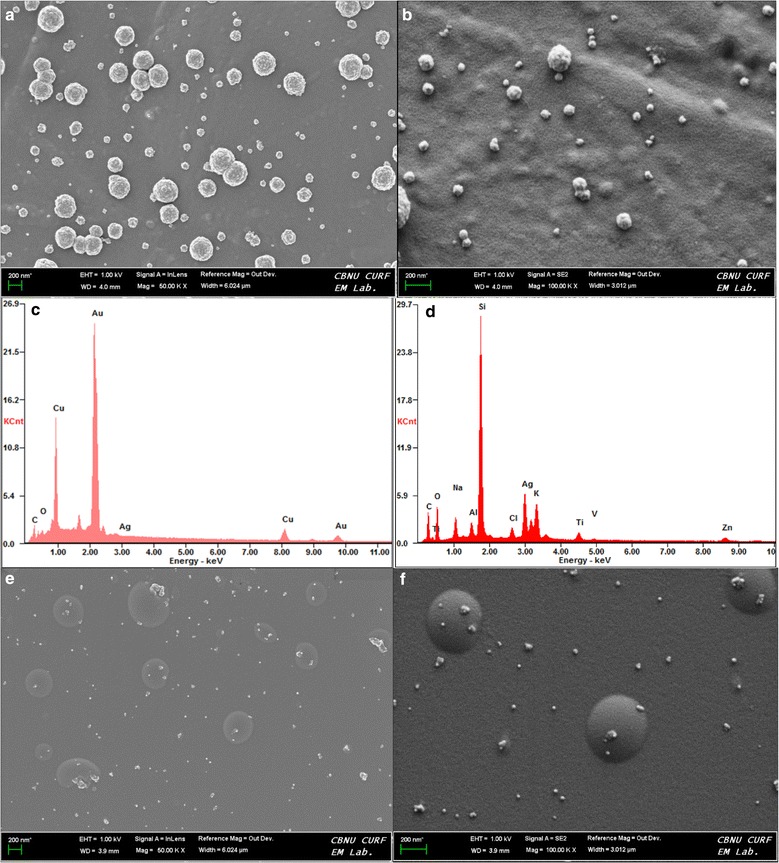
FE-SEM and EDX images of the AgNPs and Au–Ag nanoparticles: **a** Sample of Au–Ag nanoparticles showing polycrystalline structure, **b** A closer view of the polycrystalline structure showing many silver nanoparticles embedded on the surface, **c** EDX data depicting the composition of the Au–Ag nanoparticles, **d** EDX data depicting the composition of the AgNPs, **e** Sample of AgNPs showing polydispersed spherical nanoparticles, **f** AgNPs showing spherical and elongated structures

The energy-dispersive X-ray spectroscopic analysis was carried out to analyze the elemental and chemical composition of the nanostructures. The samples after drying in a hot air oven, on a copper grid/glass slide, was coated with osmium for EDX analysis. The spectrogram for AgNPs show peaks for carbon, oxygen, silicon and silver (Fig. 3d). The silver peak is comparatively very small to the silicon peak. This may be because of very small particle size of silver and low density per area, which gives more exposure to the glass slide. Whereas the Au–Ag nanocomposite sample spectrogram show peaks for carbon, oxygen, copper, gold and silver (Fig. 3c). In this case the gold peak is very high as compared to silver and copper. This suggest a very high concentration of gold and the formation of a network of interconnected particles, which is an indicant of a nanocomposite formation. This nanocomposite masks the copper grid thus a small peak of copper compared to gold. The very small peak of oxygen in Au–Ag nanocomposite sample as compared to AgNPs sample, Figs. 3c and 3d, may also suggest the utilization of oxygen for oxidation of silver, thus producing very small particles of silver in the Au–Ag nanocomposite. There was no evidence of chlorine and nitrogen in the spectrogram, which suggest no formation of Cl or N associated compounds.

**Fig. 4:**
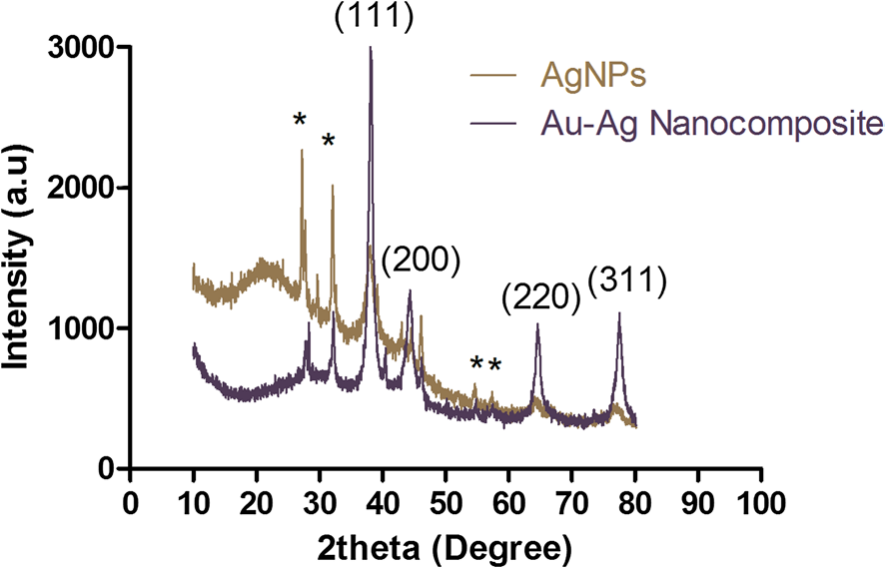
XRD showing the comparative spectra of AgNPs and Au–Ag nanocomposite sample

The X-ray diffraction pattern analysis was used to evaluate the crystallinity of the synthesized AgNPs and Au–Ag nanocomposites (Fig. 4). The peaks are indexed to (111), (200), (220) and (311) sets of the lattice planes of the face-centered cubic structure. There are also some unassigned peaks marked with a star (Fig. 4). These peaks may be assigned to the crystallization of the organic material in the bark extract [35]. The peaks of the Au–Ag nanocomposites is seen to be relatively more intense, which could be a sign of better crystallization of the material and saturation of the compounds in the organic material around the particles. This is in accordance to the aforementioned FT-IR analysis.

### Microscopic analysis

**Fig. 5:**
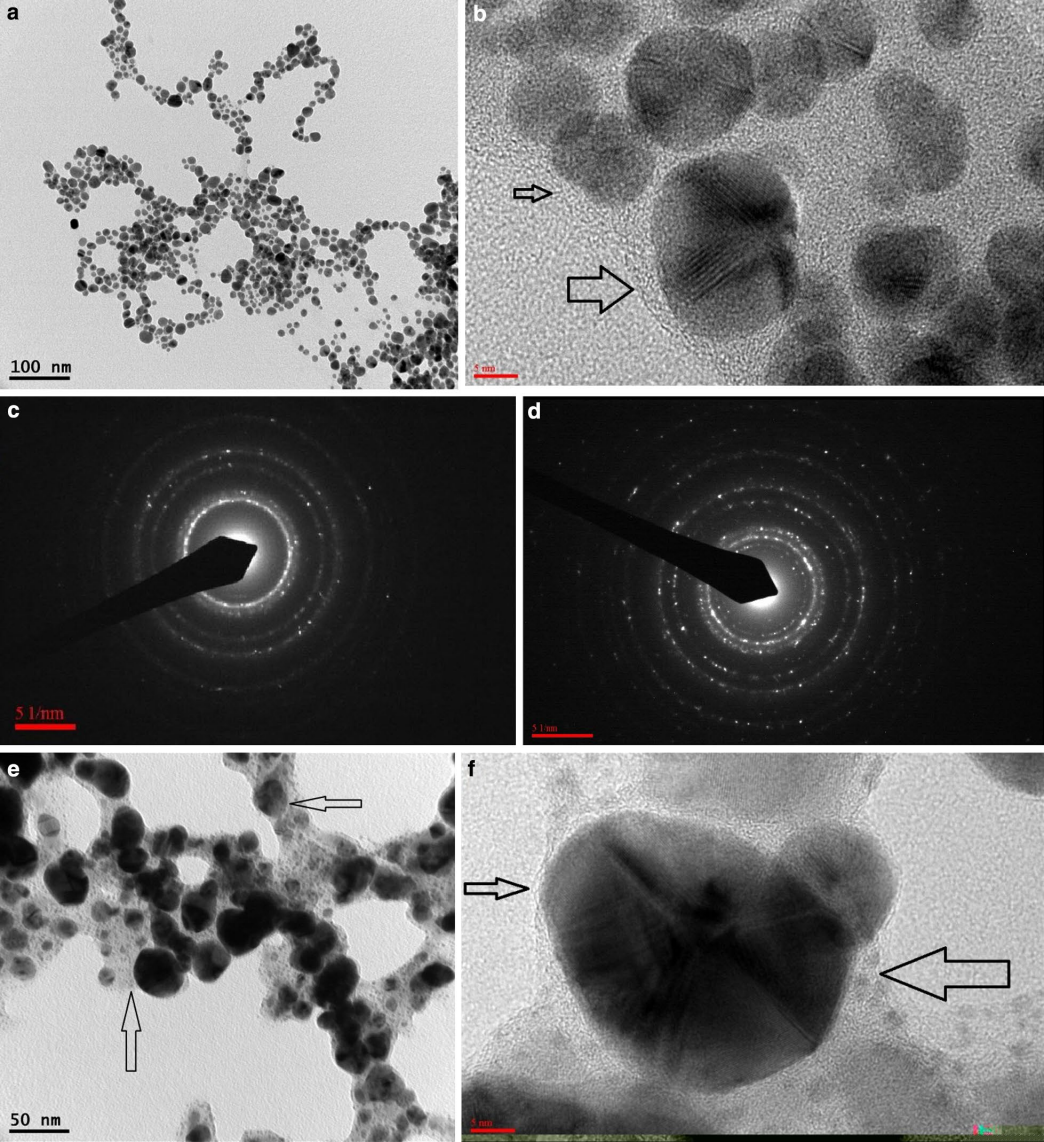
TEM images of AgNPs and Au–Ag nanocomposite specimen: **a** Polydispersed spherical AgNPs, **b** Organic material encapsulation of AgNPs, **c** Selected area diffraction pattern of AgNP, **d** Selected area diffraction pattern of Au–Ag nanocomposite particle, **e** Au–Ag nanocomposite showing polymorphic crystallization of Au particles surrounded by very small AgNPs, **f** Organic material encapsulation of Au–Ag nanocomposite

The synthesized AgNPs and Au–Ag nanocomposite were coated on copper grids and were observed under a transmission electron microscope(TEM). The photographs representing the electron microscopic studies, as shown in (Fig. 5), gives a polydispersed picture of the synthesized particles. The AgNPs were predominantly spherical in shape with a size range of 5–30 nm. The average size of the nanoparticles was found to be ~20 nm. A keen inspection of the particles revealed a shadowy layer around the particles (Fig. 5b). The speculation is that the layer is formed of the organic material in the bark extract. This kind of organic layer or capping material has been seen in previous reports of green synthesis of nanoparticles [36]. Selected area electron diffraction pattern of the silver nanoparticles show rings depicting the structure of crystalline silver nanoparticles (Fig. 5c). The Au–Ag nanocomposite, under TEM, displayed a diversity in crystal size, shape and structure (Fig. 5e). The rapid reaction of auric chloride with silver nanoparticles under limiting reductant concentration (plant extract) and reducing temperature resulted in spherical, hexagonal and elliptical shape gold particles in 15–80 nm size range. Some elongated structures could also be seen, which may be due to the agglomeration of the particles. This kind of polymorphic crystallization confirms the reaction between auric chloride, silver nanoparticles and plant extract. The images also confirm the presence of very small particles (1–3 nm) in the vicinity and over the surface of the Au particles, which may be due to the oxidation and galvanic replacement of silver nanoparticles (Fig. 5e). The galvanic replacement reaction causes the leaching of Ag^+^ ions, from the surface of the AgNPs, which in-turn reacts with the reductant in the medium and form very small AgNPs [33]. There is no formation of small AuNPs because silver is more reactive than gold and the plant based reductant is also specific to silver ion reduction. Unlike the case of AgNPs, the Au–Ag nanocomposite showed a clear existance of an organic layer as a capping material around the particles (Fig. 5f). This further confirms the findings of the FTIR characterization, which also says that the nanocomposite encapsulation is better defined than the AgNPs. The selected area electron diffraction pattern of the nanocomposite also show rings depicting the structure of crystalline gold and silver particles (Fig. 5d).

The nanoparticle and nanocomposite solutions were dropped on copper grids/glass slides and was dried in a dry air oven and was osmium coated for field emission scanning electron microscopy(FE-SEM) (Fig. 3). The FE-SEM was required to analyze the surface of the synthesized nanostructures and to further confirm the shape and size of the nanostructures (Fig. 3). The AgNPs concentration was found to be very less as cofirmed by the EDX data (Fig. 3d). The FE-SEM data also depict a predominantly spherical AgNPs with some deviations in the form of elongated particles (Figs. 3e, 3f). These elongated particles were formed mainly due to agglomeration of some silver nanoparticles. The Au–Ag nanocomposite displayed more of a polycrystalline structures (Fig. 3a). As shown in Fig. 3a, the nanocomposite specimen showed various size (50~200 nm) of spherical structures. A keen observation of the structures and the surface pattern revealed that it was composed of many nanoparticles (Fig. 3b). The size of the particles on the surface indicate towards the silver nanoparticles embedding (Fig. 3b). This could have happened due to the bonding of positively charged Ag ion onto the surface of AuNPs (Carrying negative charge) formed during the displacement and oxidation reaction (Previously discussed in the synthesis dynamics section). The EDX data also show a very high concentration of gold and very low concentration of silver and carbon (Unlike the case of silver nanoparicles where carbon concentration was very high), which could indicate that the carbon in the organic material was utilized in the formation of the spherical structures for binding the particles together.

### Antimicrobial analysis

**Fig. 6:**
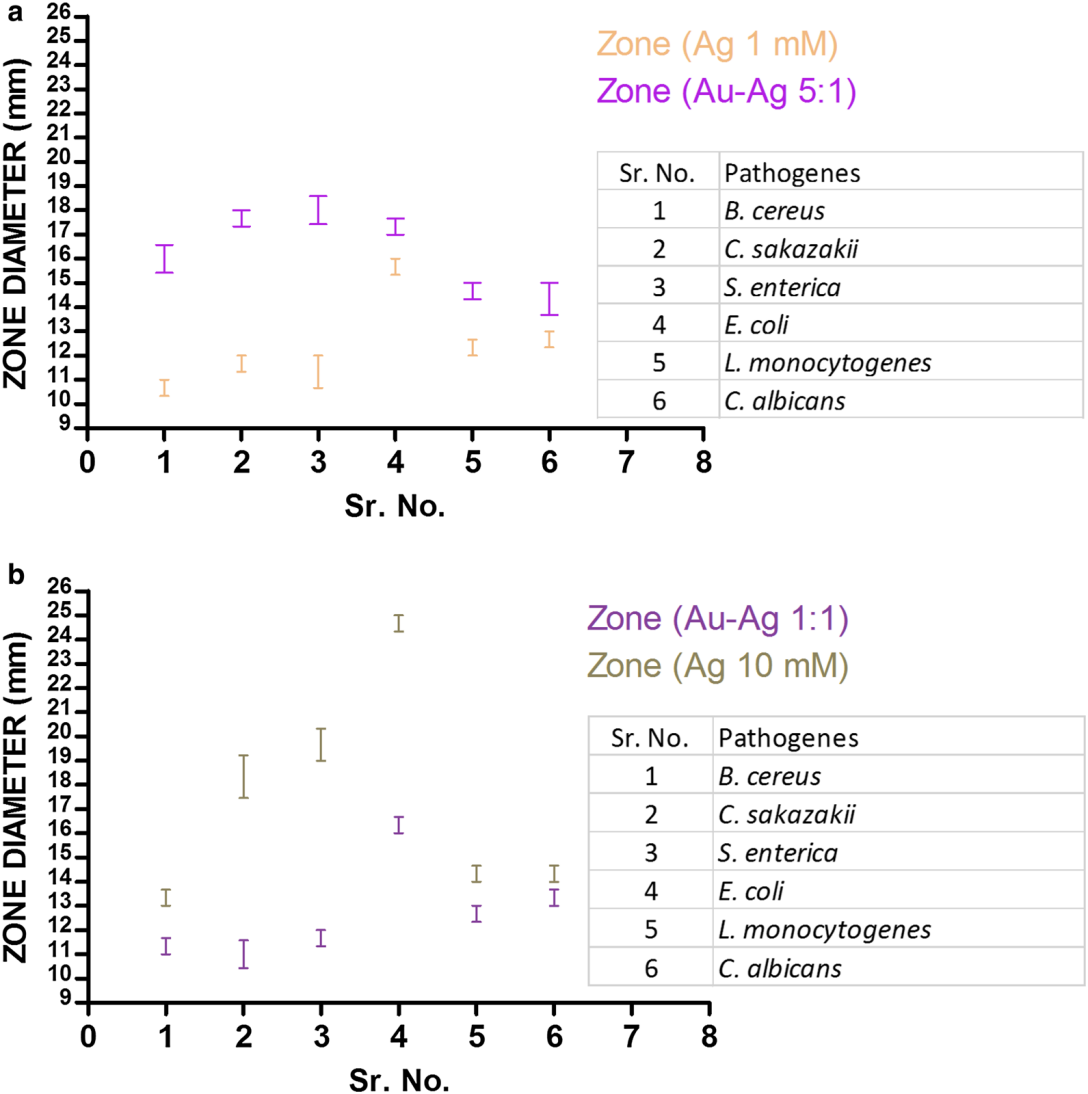
Disk diffusion analysis showing the zone of inhibition of the AgNPs and Au–Ag nanocomposite specimens: **a** Standard error of mean (SEM) of zone of inhibition by specimen of AgNPs (1 mM AgNO_3_) and Au–Ag nanocomposite (1:5 composition), **b** Standard error of mean (SEM) of zone of inhibition by specimen of AgNPs (10 mM AgNO_3_) and Au–Ag nanocomposite (1:1 composition)

The nanoparticle and nanocomposite after synthesis and characterization were analysed for their antimicrobial ability on various food and water born pathogens. The pathogenes included gram positive, gram negative and a yeast species. The nano solutions used for the antimicrobial assay included AgNP specimen containing 10 and 1 mM AgNO_3_ and Au–Ag nanocomposite specimen containing 5 and 1 mM AuCl_3_. The results of the primary antimicrobial analysis of the nano specimens using disk diffusion method is represented in (Fig. 6). A comparative ananlysis of the zone of inhibition showed that in case of *Bacillus cereus* Au–Ag nanocomposite with 5:1 composition exhibited a zone bigger than the other specimens, whereas in case of *Cronobacter sakazakii*, *Salmonella enterica* and *Escherichia coli*, AgNP with 10 mM concentration exhibited a relatively bigger zone. Au–Ag nanocomposite (5:1) and AgNP (10 mM) showed similar size of zone in case of *Listeria monocytogenes* and *Candida albicans*.

As shown in Table 1, the MIC value of Au–Ag nanocomposite (5:1) and AgNPs (10 mM AgNO_3_), against test pathogens, were in the range of 0.39–6.25 % and 0.39–3.12 %, respectively. While in the case of the nanocomposite the MIC value was relatively less for gram negative test pathogens as compared to gram positive pathogens, the AgNPs showed almost equal effect on both gram positive and gram negative (with the exception of *Cronobacter sakazakii*) (Table 1). In the case of *C. albicans*, Au–Ag nanocomposite was found to have a high MIC value (6.25 %) as compared to the AgNPs (0.78 %). This show that the Au–Ag nanocomposite (5:1) is equally effective against the test pathogens as compared to the AgNPs (10 mM AgNO_3_), with some exceptions.

**Table 1:** MIC values of AgNPs and Au-Ag nanocomposite against common food and water born pathogens

Pathogenes	MIC (AgNPs)	MIC (Au–Ag nanocomposite)
*B. cereus*	0.78	1.56
*C. sakazakii*	3.12	1.56
*S. enterica*	0.39	0.78
*E. coli*	0.39	0.39
*L. monocytogenes*	0.78	0.78
*C. albicans*	0.78	6.25

The above results show that Au–Ag nanocomposite (With high gold to silver ratio) is more effective antimicrobial than the AgNPs. This may be due to the very small size AgNPs present around the Au particles in the Au–Ag nanocomposite, which increases the surface area of the nanocomposite and thus enhance the antimicrobial activity.

## Experimental

### Plant extract

The bark of *M. azedarach* was collected from a private nursery in state of Odisha, India. The bark was shredded into medium size pieces and was kept for drying under shade in room temperature. After drying for about 20 days the pieces were made to a powder form. 1 g of the *M. azedarach* bark powder was then mixed into 100 ml deionized water and was sterilized in an autoclave for 20 min at 121 °C and 15 psi pressure. After autoclaving the mixture was cooled to room temperature under UV. This sterilization process is required to eliminate fungal contamination. After cooling the mixture is filtered using a Whatman 2 filter paper into a sterilized container and is stored for further use.

### Nanostructure synthesis

The filtered bark extract of *M. azedarach* thus obtained was used as a source of reductant for the nanostructure synthesis. A stock aqueous solution of 1 M AgNO_3_ and 1 M AuCl_3_ was prepared. The silver nanoparticles were synthesized by adding 1 M silver nitrate (AgNO_3_) stock solution into separate glass tubes already containing 10 ml of the plant extract to make final concentrations of 1, 5, 10, 15 mM, respectively and was autoclaved for 1 min at 121 °C and 15 psi pressure. Simultaneously, a set of 1 mM AgNO_3_ with plant extract was autoclaved for 5 min with same conditions to compare the time for complete synthesis. The synthesis of Au–Ag nanocomposite is a two-step process. In the first step, 1 M AgNO_3_ stock solution is added into separate glass tubes already containing 10 ml of the plant extract to make final concentration of 1 mM each and was converted to AgNPs by autoclaving the mixture for 1 min at 121 °C and 15 psi pressure. In the second step, the pressure was released and immediately 1 M AuCl_3_ was added, to the hot AgNPs solution tubes, to make final concentrations of 1, 5, 10 and 15 mM. Then the tubes were vortexes and were allowed to cool down in dark at room temperature. The nanoparticles and nanocomposite thus prepared were stored at room temperature for further characterization and analysis.

### Nanostructure characterization

The nanoparticles and nanocomposite thus synthesized were passed through a series of techniques to prove the authenticity of their quality, quantity and to understand the nanostructure dynamics in the aqueous extract.

Preliminary characterization of the synthesis of nanoparticle and nanocomposite was done by UV-visible spectral analysis, of the synthesized solutions, using the Epoch microplate spectrophotometer, BioTek Instruments Inc. The analysis was done by taking 100 µl of the nanoparticle and nanocomposite solution sample in a 96-well microplate and scanning it within 300–800 nm wavelength. Then the best concentration was chosen, for further characterization, based on the analysis of the spectral peaks obtained from the scan. The concentrations showing best peaks were analyzed for their elemental concentration (Concentration of Au and Ag) by inductive coupled plasma mass spectrophotometer (ICP-MS) model 7500a, Agilent technologies.

Then the nanostructure solutions with the best concentration were analyzed by FTIR spectroscopy to determine the functional groups involved in the nanostructure synthesis and stabilization. The nanoparticle and nanocomposite solutions were concentrated, dried and powdered. The dried powders were pellet out in KBr pelletizers using Perkin Elmer model spectrum GX operated at a wavelength of 350–4500 cm^−1^ at a resolution of 0.4 cm^−1^ with the wavelength accuracy of 0.1 cm at 1600 cm^−1^.

Then the nanoparticles and nanocomposite were coated on carbon-coated copper grids (400 mesh) and was observed under a transmission electron microscope (Orius SC10002 JEM-2010) for their shape, size and structure. The grids were dried and coated with osmium and was further observed under FE-SEM (S-4800, Hitachi, Japan). The elemental analysis was done by the energy-dispersive X-ray spectroscopy (S-4800, Hitachi, Japan) to determine the relative composition of the specimen.

The powdered samples of the nanoparticles and nanocomposite were packed onto the XRD grids and spectrograms were recorded by using a multipurpose high performance X-ray diffractometer (X’pert powder, PANalytical; The Netherlands).

### Antimicrobial activity analysis

After multi-step characterization of the synthesized nanostructures, the nanoparticles and nanocomposite solution showing the best characteristics were scrutinized for their antimicrobial effects.

The preliminary antimicrobial activity assay was done by disc diffusion assay techniques. In this assay 20 ml of the suitable agar medium, for each organism, was layered on petriplates and was allowed to set and cool. Then 100 µl of the cultured microorganisms (10^7^ CFU/ml concentration) were mixed into 5 ml softagar and was overlaied on top of their respective agar medium plates. After the plates cooled down the discs were put on them. Then 50 µl of the test nanostructure solutions were dropped on the discs. The plates were incubated for 12 h at 37 °C, before calculating the zone of inhibition. All the assays were done in triplett and the standard error of the mean (SEM) of the zone of inhibition was plotted on to the graph for analysis (Fig. 6).

The test nanoparticles and nanocomposite solutions which showed best zone of inhibition in the disc diffusion assay were further evaluated for their minimum inhibitory concentrations (MIC). The MIC evaluation was done on a 96 well plate. 200 µl of the nanostructure solution was pipette into the six wells (leaving the first and the last well) in column 1 (far left side of the plate). Then the wells in each row were filled with 100 µl of broth medium suitable for the growth of each organism. After that 100 µl of the nanostructure solution was taken from column 1 and was serially diluted along the row until column 10. Then 5 µl of the the microorganisms were inoculated into each wells containing their respective medium except column 12, which served as blank. Then 200 µl of sterile water was pipetted into the wells in row 1 and row 8 of the plate (To prevent the wells from drying). Then the 96-well plate was incubated at 37 °C for 24 h. After incubation 5 µl from each wells were inoculated on agar medium plates and the plates were incubated for 24 h. After that the plates were studied for growth or no growth and the wells containing the minimum concentration of test solutions, showing no growth, were declared as the MIC. The MIC values were tabulated in terms of percentage concentration (The concentration of test solutions in column 1 was considered as 100 %) (Table 1).

## Conclusion

The strategy employed in this study clearly proves the hypothesized hydrothermal acceleration of activity of the plant based reductant in synthesis of AgNPs and Au–Ag nanocomposite. We found that the nanostructures not only could be synthesized rapidly but also could be synthesized at high concentrations. Although nanoparticles are being synthesized using chemical and physical methods, however, the adverse effects of these methods sought for a more sustainable and stable method for synthesis of nanostructures. The advantage of nanostructure synthesis using autoclave technology is that the nanostructures have the same composition, structure and property in all batches of production. This kind of stable and ecofriendly production is only possible due to the enclosed and controlled environment of the autoclave. The dynamics of the metal salts and plant extract was explained using chemical equations which were in accordance to the characterization of the synthesized nano solutions. The synthesized nanocomposite and nanoparticles were found to be very effective against the common food and water born pathogens. The antimicrobial effectiveness of the synthesized Au–Ag nanocomposite, with high gold to silver ratio, reduces the dependency on the AgNPs, which has been found to be environmentally more toxic than the gold counterpart. We hope that this new strategy will change the present course of green synthesis. The rapidity of synthesis will also help in industrial scale green production of nanostructures using *M. azedarach*.
